# Supplementary material for: Radiochemical Feasibility of Mixing of 99mTc-MAA and 90Y-Microspheres with Omnipaque Contrast
Source: Molecules. 2022 Nov 7;27(21):7646. doi: 10.3390/molecules27217646 (PMC9656951; doi:10.3390/molecules27217646)

Supplementary materials

For

Radiochemical Feasibility of Mixing of ^{99m}Tc-MAA and ^{90}Y-Microspheres with Omnipaque Contrast

Chang-Tong Yang[1,2], Pei Ing Ngam[1,3], Vanessa Phua Jing Xin[1], Sidney Wing Kwong Yu[1], Gogna Apoorva[2,4], David Chee Eng Ng[1,2]*, Hian Liang Huang[1,2]*

1 Department of Nuclear Medicine and Molecular Imaging, Radiological Sciences Division, Singapore General Hospital, Outram Road, Singapore 169608, Singapore

2 Duke-NUS Medical School, 8 College Road, Singapore 169857, Singapore

3 Department of Diagnostic Imaging, National University Hospital Singapore, 5 Lower Kent Ridge Road, Singapore 119074, Singapore

4 Department of Vascular and Interventional Radiology, Radiological Sciences Division, Singapore General Hospital, Outram Road, Singapore 169608, Singapore

Figure S1. Microscopic images of sample **1**, **2**, and **4**.

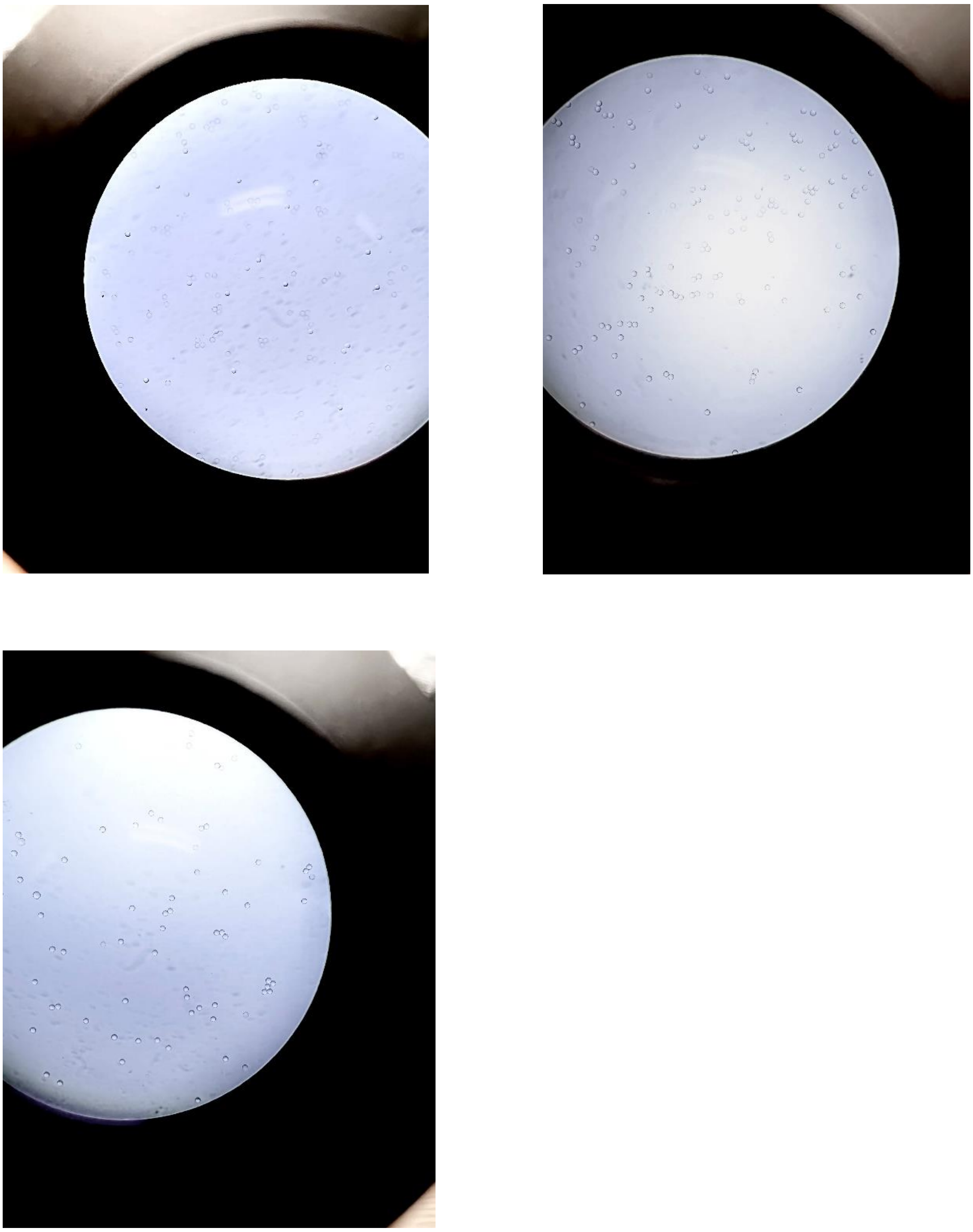

Figure S2. TLC chromatograms of ^{99m}Tc-MAA in contrast omnipaque with same volume of serum incubated at 37°C at 1 hour and 3 hours, respectively. (Left) 1h, (Right) 3hs.

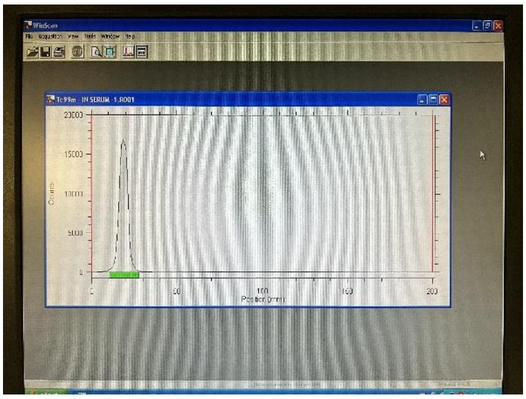

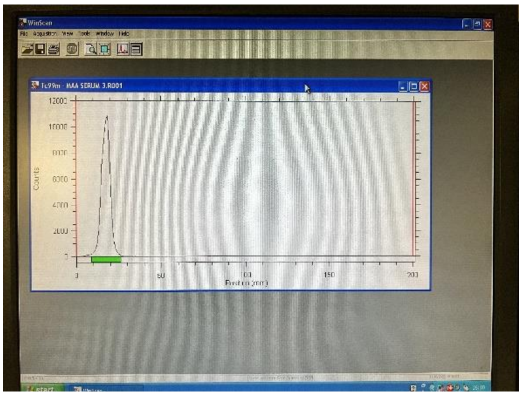

Supplement: Supplementary file 1 [file molecules-27-07646-s001.zip › molecules-1981927-supplementary.pdf]
